# Editorial

**Published:** 1875-04

**Authors:** 


### Editorial.

### *"Bills of Interest to the Medical Profession."*

Under this caption, the Philadelphia *Medical Times* editorially directs attention to three "Bills" now pending before the Legislature of the State of Pennsylvania. Of the first, "An Act to Regulate the Practice of Medicine, Surgery and Obstetrics in the Commonwealth of Pennsylvania," we will say, in the language of our excellent cotemporary, "we think this bill a very good one, although, of course, we should like to have seen it go further," so as not only to "do away with two great evils —practicing medicine without a diploma, and peripatetic quackery," but also to accomplish the organization of a "State Board of Examiners, before whom every aspirant for practice should appear." We should like very much to see statutes enacted in this State which should comprehend such desirable objects.

"The second act," however, is that which has more especially attracted our attention, and the strong resemblance in its features to one of a similar character which was urged upon the Legislature of this State at its last session, and to another, also, which was urged upon Congress at its last session, in the guise of a National Board of Health, suggests a common paternity for each and all of them. The Philadelphia *Medical Times* is generally acute of perception and merciless to frauds of all sorts, and if, after a critical analysis of this "Bill," a humbug and fraud is not detected, then we will acknowledge that we have mistaken the "foot-prints in the sand," and that it is not *our* African hidden in our neighbor's wood-pile, but one of his own, who closely resembles him. There is a German proverb: „Wo Rauch ist, da ist auch Feuer,"—hence our suspicions.

This Journal, under the caption, "An Asylum for Imbeciles," directed public attention to the true gist of this same "Bill," for we shall be much surprised if it be not the same Bill, or an "artist's proof" of it, as presented to the Legislature of the State of Illinois, showing what an admirable contrivance was therein devised for the creation of a sinecure. The essence of the whole matter being a "Secretary," endowed with a position of legal—if not of medical—respectability, a modicum of superficial acquirements to dazzle country members, a panoply of impenetrable brass, and "sine qua non"—"three thousand dollars a year." Does the State of Pennsylvania contain "five physicians" fitted to fill the position designed for them by this "Bill," willing to serve without salary? If so, she can boast of five true philanthropists, ready to sacrifice themselves for the—support of the Secretary. Has the State of Pennsylvania a physician fitted, by the possession of great administrative ability, a liberal education, profound and extensive scientific attainments, studious and laborious habits of life, to fulfill the important duties which must devolve upon the Secretary of the State Board of Health of that vast Commonwealth, willing to sacrifice the splendid professional position and lucrative practice which such qualifications always command, for the paltry stipend of three thousand dollars? If so, she must be credited with another philanthropist, and may rejoice in her possessions over her sister States—certainly over ours, who have none such. We had one once, but he has departed, and the places which knew him now know him no more, and we know not whither he has gone, unless the Commonwealth of Pennsylvania has borrowed him, or he has vanished in "smoke."

To our friend the *Times* we are under many obligations for its skillful detection and brave denunciation of many frauds of many kinds, and we would willingly cancel some of them by a word of caution. Look well to your "Bills" before they become laws, or you may find too late that you have permitted a parasite, the shae "Secretary," to secure a legal hold upon the body professional, and attract to it as much odium as would the presence of a parasite of another kind upon the body personal. H.

### *Medical Education and Medical Legislation.*

The period of the annual recurrence of Medical College Commencements is always the occasion for diatribes, in medical and secular journals, upon the defects of "the Modern System of Medical Education," the "low standard of preliminary acquirements," the "superficial character of the instruction imparted to medical students," and various other disadvantages which the American, and especially the Western, medical student is supposed to sustain, in comparison with his more fortunate compeer on the other side of the ocean. The stereotyped facetiæ about "crops of sawbones," "licenses to kill," etc., occupy their usual places in the "funny columns" of the press. The critics are not, however, altogether merciless, for while indicating the faults they are charitable enough to designate the remedy, and this they find in that great panacea of the untutored mind, "the law." The grand catholicon, under whose magical influence all these evils are to disappear, is "Medical Legislation." Teachers are to be made competent and conscientious, students instructed and accomplished, physicians learned and skillful, by acts of legislature.

Lord Thurlow said that an "act of Parliament could not be drawn through which he could not drive a coach and horses." Might not a medical student be driven through an "Act of Legislature"? Legislation, then, appears to be, in the eyes of the reformers, the means, and the only means, by which the medical profession is to be brought "up to the level of the legal and clerical," with each of which it is now most discreditably compared.

Having had much better opportunities for judging this question than the majority at least of the critics and reformers, from both the outside and inside point of view, we will take the liberty to examine the subject a little more closely. The relative qualifications of the three so-called learned professions may be compared in a very few words. The first, the theologians, may be left to themselves, as of the three hundred and sixty-five (or seven, we forget which,) sects, no one can be found unwilling to condemn all the others of gross ignorance, and with some measure of justice, as but one can be right. The qualifications of the members of the legal profession are exemplified in the almost impossibility of securing the conviction of many criminals of whose guilt there is a moral certainty, Moreover, the attenuation of an estate emerged from chancery is aptly comparable to the emaciation of a patient convalescent from a wasting disease ; and while it is true in this and some other communities, that the legal profession opens more numerous collateral avenues to wealth, into which many of its members have entered, than the medical, it is equally true that, in regard to professional education, general culture, and practical skill, the members of the latter are fully equal to those of the former, class for class, rank for rank. The comparison is confidently challenged, without fear of the result.

To the self-styled critics, those of them at least who are members of a profession which they seem anxious to decry, a question is in order. Is the low standard of professional education which they deplore that to which they themselves were brought, or that to which the student of to-day must conform ?

We assert boldly, that were the great mass of general practitioners required to pass the examination to which medical students are now subjected, and which is yearly becoming more rigid, the percentage of rejections would be far greater than in an average class of candidates for graduation in many of the medical colleges. Don't condemn the graduate of to-day until you have measured your acquirements with his. All that is old is not venerable. There are antique as well as modern shams, and the "....laudator temporis acti" lived too in the days of Horace.

In the opinion of many, these evils may all be effectually remedied by the establishment of a State Board of Examiners, whose certificate should be not only *prima facie* evidence, but proof of the competency of medical men. The idea is plausible and superficially captivating, but will it justify our endorsement when critically analyzed? In the first place, we fail to perceive in what respect the certificate of a State Board of Examiners will be more positive evidence of competency than the diploma of the faculty of a college, as the latter would be, in all probability, the larger body of the two, and equal at least to the former in professional standing, being composed of physicians deemed by their colleagues—the most competent tribunal—peculiarly fitted for their positions.

Faculties of medical colleges are always, to say the least, up to the average of their profession in point of ability and professional acquirement, and usually above it, never below. Can the same be said of "Boards" consisting in whole or in part of medical men?

How could these Boards be appointed? by the Governor? that is to say, by personal influence; by the Legislature? that is to say, by political influence; or by the people? that is to say, by popular influence. Than the first of these there could hardly be a more unreliable method of choosing the fittest, except the second and third, and the same might be said of each of the other methods respectively. The Governor would appoint his own physician and his friends, or suffer an imputation upon his own judgment. The Legislature would appoint those having most political influence, in the acquisition of which no man ever became an accomplished physician. And the people would elect the most popular doctors, who would generally be found to have cultivated popularity, to the exclusion of science.

Considered in its economical aspects, the scheme presents features demanding careful scrutiny in these days of high taxes and public and private indebtedness. A "State Board" of Medical Examiners must consist of at least one expert in each department of medicine, as now taught, with provision for the addition of others as they may become necessary for the advancement of science, *i. e.*, in Anatomy, Chemistry—organic and inorganic—Materia Medica and Medical Botany, Physiology, Pathology, Surgery, Obstetrics and Gynæcology, Practical Medicine in its various branches, Microscopy, Electrology, etc. It would be difficult to constitute a Board of less than ten members expert in all these departments of medical science and art. This is only what every medical college aims to accomplish—and, it would appear, with a certain measure of success—for its own students. Moreover, this Board must occupy itself exclusively with its duties and the preparation for them, and must be paid a minimum of ten thousand dollars for each of its members. Add to this fifty per cent. for incidental expenses, and we have an item of one hundred and fifty thousand dollars to be added to the annual taxes of the people of the State. How will this view of the picture please the people who clamor so loudly for "medical reform"?

In this country, for the last ninety-nine years, we have been playing the game Republic, in which "we make believe" that every player has equal rights to "life, liberty, and the pursuit of happiness." It is true the game is nearly played out, but some of us are not yet tired, and want to "play on till dark;" and while others may clamor to give up and play a new game, perhaps "Monarchy," or "Military Dictatorship," or something else, we insist upon the maintenance of the original conditions. Some of us find our pursuit of happiness in the care of bodies, some in the care of souls, and some in the care of estates. Because one may not save some vile body from the grave by arts of which people know little, shall he be forbidden by the law from trying to save others? Because one may not save some viler souls from hell by arts of which the people know less, shall he be forbidden by the law from trying to point out the other road, although his knowledge thereof be none of the clearest? Because one fails to convince an unimpressible jury that the blood-stained fingers of his client in the dock are whiter than the driven snow, and thus save his neck from the gallows, shall he be forbidden by the law to try to pluck its legitimate fruit from that too barren tree?

This is "paternalism" run mad. If the State requires an arbitrary standard of proficiency in one rank of life, it must exact it similarly in all. There must be a State Board for the examination of clergymen, doctors, lawyers, apothecaries, grocers, liquor dealers, horse-shoers, plumbers, sewer builders, and night scavengers; for they and many more are directly instrumental in the preservation of public—and private—health.

Again, if the State exacts an arbitrary standard of professional proficiency, it is obligatory upon the State to provide the means and appliances whereby it may be reached, otherwise the State becomes an irresponsible tyranny.

This involves the establishment of a system of public education, comprehending every branch of human knowledge, which must be free to all, since all are compelled to accept its provisions. Have the reformers ever counted the cost of so comprehensive a scheme?

We have seen several of these projects for the establishment of "State Boards," and—while we have no desire or intention to stigmatize all as such—these were all nothing more nor less than contrivances by which somebody, unable or unwilling, or both, to attain professional position by hard work, sought to acquire some temporary notoriety, a small stipend, and "the chances," at the expense of the State.

In conclusion, we assert our most thorough sympathy with those who desire the elevation of the standard of professional education, but cannot endorse all the utopian schemes which have been devised for the accomplishment, believing the only reasonable plan—which must eventually succeed—to be the silent force of example.

Let every critic and reformer study diligently and faithfully to become truly expert in some department of his profession, and all who come within the range of his influence will be stimulated to follow his example—and so the standard of medical education will be raised *tuto, cito, et jucunde.* H.

